# Inactivation of Viruses by Charged Cinnamaldehyde Nanoemulsions

**DOI:** 10.3390/foods14060931

**Published:** 2025-03-09

**Authors:** Pragathi Kamarasu, Minji Kim, David Julian McClements, Amanda J. Kinchla, Matthew D. Moore

**Affiliations:** Department of Food Science, University of Massachusetts, Amherst, MA 01003, USA; pragathikamarasu@gmail.com (P.K.); or smileminji0123@gmail.com (M.K.); mcclements@foodsci.umass.edu (D.J.M.); amanda.kinchla@foodsci.umass.edu (A.J.K.)

**Keywords:** foodborne viruses, norovirus, human coronavirus, nanoemulsions, cinnamaldehyde oil

## Abstract

Viral pathogens are a considerable public health burden, and so inactivating viruses in the environment is critical. This study compared the antiviral activity of cinnamaldehyde nanoemulsions (CNE) and cinnamaldehyde oil (CNO) on a non-enveloped norovirus surrogate bacteriophage (MS2) and an enveloped human coronavirus 229E (HuCoV-229E). MS2 bacteriophage and HuCoV-229E were treated with different concentrations of CNE or CNO (0.5–3.5%). After treatment for 1 h, the reduction in MS2 numbers was significantly less for the CNE than for the CNO. For instance, the log reductions in MS2 numbers were 4.02 ± 0.10 and 2.78 ± 0.34 PFU/mL after treatment with 3.5% and 0.5% of CNO, whereas they were only 1.54 ± 0.08 and 0 PFU/mL after treatment with the equivalent CNE, respectively. Conversely, a significant reduction in HuCoV-229E was observed for the nanoemulsion-based treatment at high cinnamaldehyde levels. Specifically, when treated with 0.5% cinnamaldehyde, there was a 1.35 ± 0.23 and 3.08 ± 0.17 log PFU/mL reduction for the CNE and CNO treatments, but when treated with 2.5% cinnamaldehyde, there was a 5.98 ± 0.12 and 4.43 ± 0.38 log PFU/mL reduction, respectively. These results suggest that the efficiency of the essential oil as a disinfectant against coronavirus-229E can be increased when it is incorporated in a nanoemulsion at an appropriate concentration. The better efficacy of the nanoemulsion formulations against coronavirus-229E than against MS2 bacteriophage may have been because the cinnamaldehyde oil droplets could penetrate into and deactivate enveloped viruses more effectively than non-enveloped ones.

## 1. Introduction

Human norovirus is the leading cause of foodborne illness globally, accounting for 200,000 deaths annually. Noroviruses are non-enveloped viruses in the *Caliciviridae* family that consist of single-stranded positive-sense RNA. They account for the majority of foodborne illnesses, as well as being the fourth leading cause of foodborne death [1,2]. A significant challenge in controlling noroviruses is their general resistance to many common inactivation agents, such as UV radiation, ethanol, and chlorine-based treatments. Studies conducted by other researchers suggest that there are only a few efficacious inactivation agents for noroviruses, many of which inactivate the viruses by oxidizing the non-enveloped protein capsid; however, the challenge faced is that none of them are food-grade [3]. Similar challenges arise with the inactivation of other viruses, such as SARS-CoV-2, which has led to widespread concern about the transmission through environmental surfaces, as well as food and packaging [4].

Although SARS-CoV-2 is structurally distinct from norovirus, both viruses share similarities in their environmental persistence and the need for effective inactivation strategies. The effectiveness of these strategies is crucial not only for foodborne illness control but also for minimizing the spread of viruses in community settings. Human coronavirus 229E is another virus in the *Coronaviridae* family with related structural features to SARS-CoV-2, which is also a human pathogen that can cause upper respiratory infections, albeit being less severe than SARS-CoV-2, but can still pose a public health burden. [1]. Additionally, human coronavirus 229E has been used as a surrogate for studying viral environmental persistence and inactivation. Like SARS-CoV-2, 229E has been demonstrated to persist in and be transmitted via environmental surfaces, making inactivation important for control [4]. Hence, this virus has been chosen as a surrogate for SARS-CoV-2 to conduct inactivation studies.

Although numerous conventional inactivation agents have been demonstrated to be effective against coronaviruses, their frequent application can leave residues that are harmful to human and environmental health [5]. Consequently, there has been interest in the development of less harmful plant-based antiviral agents such as the bioactive phytochemicals found in essential oils (EOs) [6]. EOs are generally recognized as safe (GRAS) by the Food and Drug Administration (FDA). They are widely used to impart flavor in numerous foods and are typically considered to be more environmentally friendly, sustainable, and healthier alternatives for antiviral applications than traditional synthetic antiviral agents. EOs contain numerous phytochemicals such as aromatic hydrocarbons, terpenes (monoterpenes and sesquiterpenes), terpenoids, esters, alcohols, organic acids, aldehydes, and ketones, and their antimicrobial activity is usually attributed to combinations of these components [7]. A major challenge faced by researchers working in this area has been to identify the specific phytochemicals responsible for their antimicrobial activity due to their chemical complexity [8].

EOs derived from the bark of cinnamon have been reported to exhibit strong antimicrobial activity, with the primary active compound being cinnamaldehyde, which comprises around 90% of the oil [9]. Indeed, cinnamon essential oils have been reported to exhibit antimicrobial, antitumor, anti-inflammatory, and antioxidant activities [10]. Researchers have reported that cinnamaldehyde essential oil (CNO) has a strong antibacterial effect against *Staphylococcus epidermidis* [11], *Staphylococcus aureus* [12], and *Aeromonas hydrophila* [13], which was attributed to cell membrane damage, leakage of intracellular contents, and polarization of the cytoplasmic content. CNO has shown promising results against bacteria and fungi; however, its antimicrobial efficacy against viruses has not been as widely explored. Fabra et al. evaluated its antiviral activity against human norovirus surrogates (murine norovirus and feline calicivirus), as well as against hepatitis A virus. However, only low levels of viral inactivation were observed [14]. The inability of CNO to successfully inactivate these viruses can be attributed to their lack of a lipid envelope, which makes them more resistant than most enveloped microbes to thermal, chemical, and environmental stresses [15].

Nanoemulsions are increasingly being used in the pharmaceutical, food, and cosmetic industries as delivery systems for antimicrobial agents. Studies have shown they can improve the dispersibility, stability, release properties, and activity of encapsulated active compounds [16]. Numerous studies have shown that encapsulating EOs or their active ingredients in nanoemulsions can enhance their efficacy against bacteria and fungi [11,17,18]. As an example, Paudel et al. reported that encapsulation of cinnamon essential oil within nanoemulsions greatly enhanced its ability to inactivate *Listeria monocytogenes* and *Salmonella enterica Typhimurium* [19]. This increase in antimicrobial activity was mainly attributed to the small dimensions of the EO-loaded droplets, which enhanced their interactions with the target cells. The efficacy of nanoemulsions can be further improved by controlling the electrical characteristics of the oil droplets they contain. For instance, the utilization of cationic droplets can increase their interactions with anionic pathogens through electrostatic attractive forces [20].

In general, viruses may or may not be coated by a lipid envelope, which would be expected to impact their interactions with EO-loaded droplets. We therefore hypothesized that the efficacy of EO-nanoemulsions as antiviral agents depends on the presence of a lipid envelope around the viruses. Norovirus has hydrophobic protein patches in the capsid that are involved in folding and maintaining icosahedral contacts that help maintain its structure [21]. The potential of nanoemulsions to deliver antiviral agents to these hydrophobic patches and disrupt capsid stability of non-enveloped viruses has not been investigated previously, nor has the efficacy of cinnamaldehyde nanoemulsions against enveloped coronaviruses. The objective of this work was, therefore, to assess the effectiveness of cationic cinnamaldehyde nanoemulsions (CNEs) and bulk cinnamaldehyde oil (CNO) as inactivation agents against enveloped virus human coronavirus-229E (HCoV-229E), a surrogate for SARS-CoV-2, as well as the non-enveloped virus MS2 bacteriophage, a surrogate for human norovirus. Cationic EO-loaded droplets were formed by using a positively charged surfactant, hexadecyltrimethylammonium bromide (CTAB), to formulate the nanoemulsions.

## 2. Materials and Methods

### 2.1. Materials

Pure cinnamaldehyde essential oil (W228613-100G-K) was purchased from the Sigma-Aldrich Company (St. Louis, MO, USA). Tween-80 and hexadecyltrimethylammonium bromide (CTAB) were obtained from BioBasic (Markham, ON, Canada). Nature’s Way Medium Chain Triglycerides (MCT oil) was purchased from Nature’s Way, Green Bay, WI, USA. Human coronavirus-229E (ATCC-VR740), MS2 bacteriophage (ATCC 15597-B1), and a host bacteria *Escherichia coli* strain C-3000 (ATCC 15597) were obtained from ATCC (Manassas, VA, USA). The HUH-7.5 cell line was obtained courtesy of Dr. Brett Lindenbach (Yale University, New Haven, CT, USA).

### 2.2. Preparation of Cinnamaldehyde Essential Oil Nanoemulsions

**Figure 1:**
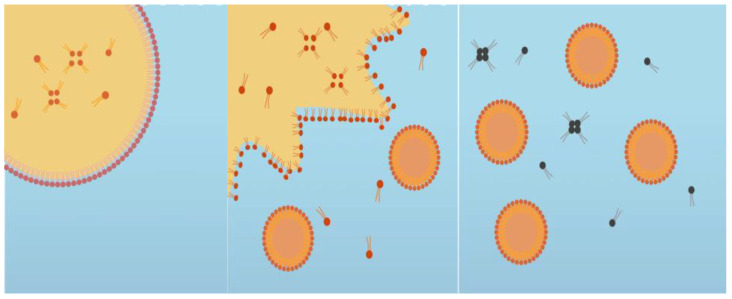
The low-energy emulsification, spontaneous emulsion method is depicted, showcasing the spontaneous droplet formation process.

**Figure 2:**
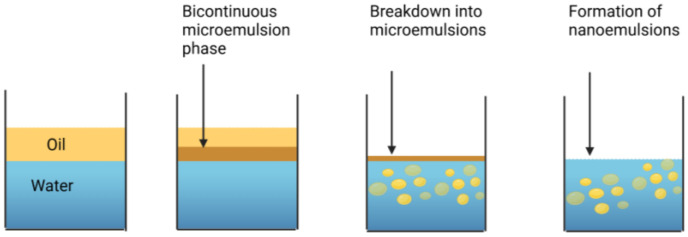
The nanoemulsion formation takes place in two stages. Initially, a bicontinuous microemulsion phase containing oil and surfactant is formed. This phase then spontaneously breaks down when it comes into contact with water, leading to the formation of a nanoemulsion containing small oil droplets coated by surfactant. This process occurs due to the generation of supersaturated regions that promote nucleation and droplet growth phenomena.

A cinnamaldehyde essential oil-in-water nanoemulsion was prepared using the spontaneous emulsification method shown in Figure 1. This was performed by homogenizing 10% *w*/*w* oil phase with 90% w/w aqueous phase. The oil phase was prepared by mixing CNO (antimicrobial oil) and MCT oil (carrier oil) at 700 rpm using a magnetic stirrer at room temperature for 5 min, and then adding a non-ionic surfactant (Tween 80) and stirring for another 3 min. The aqueous phase was prepared by dissolving cationic surfactant (CTAB) in distilled water. The spontaneous emulsification process was performed by slowly adding 10% of the oil phase to 90% of the aqueous phase while continuously stirring at 700 rpm for 30 min using a magnetic stirrer (Figure 2) [22]. The impact of varying the essential oil, surfactant, and carrier oil concentrations on the characteristics of the nanoemulsions was examined. The composition that resulted in an optimized particle diameter (around 100 nm), zeta potential (around +25 mV), and polydispersity index (0.2–0.3 PDI) was chosen to conduct the antiviral studies. In addition to the nanoemulsion, antiviral analysis was also conducted with plain CNO suspended in water. 

### 2.3. Shelf-Life Study of the Cinnamaldehyde Nanoemulsion

Following their fabrication, the stability and physicochemical characteristics of the nanoemulsions were analyzed. The nanoemulsions were stored at different temperatures (4, 20, and 37 °C) for periods ranging from 0 to 28 days and their properties were analyzed on days 0, 1, 3, 7, 14, 21, and 28. A particle electrophoresis/dynamic light scattering instrument (Zetasizer Nano ZS, Malvern Instruments, Malvern, UK) was used to analyze the zeta potential, mean diameter, and polydispersity index of the oil droplets in the nanoemulsions. Prior to analysis, each sample was diluted with a 1:100 ratio of deionized water. Visual assessment was employed to analyze the separation of the different components in the nanoemulsion during storage. Stability measurements were carried out in triplicate.

### 2.4. Antimicrobial Activity

#### 2.4.1. MS2 Bacteriophage

The antiviral activity of the CNO and CNE samples was tested against MS2 bacteriophage, which is a commonly used surrogate for human norovirus. *Escherichia coli* strain C-3000 was used as a host to propagate MS2 bacteriophage. The number of bacteriophages in plaque-forming units per milliliter (PFU/mL) was determined by the overlay agar plating method [23]. Briefly, tryptic soy broth (TSB), 0.5% tryptic soy agar soft media, and 1% tryptic soy agar (TSA) plates were prepared. The propagation and purification of bacteriophage were as follows: *E. coli* was inoculated into TSB and placed in an incubator on a shaker at 100 rpm until it reached an optical density of 0.6 cm^−1^ measured at 600 nm using a UV-visible spectrophotometer (V-1200, VWR, Radnor, PA, USA). Next, a suspension assay was conducted for different reaction times by using a neutralizing buffer (3% beef extract) and varying concentrations of both the CNO and CNE disinfectant against the phage. This was followed by serially diluting the MS2 bacteriophage and adding 700 μL of the inactivation agents, 300 μL of *E. coli*, and 36 μL of the supplements (0.1% glucose, 2 mM CaCl_2_, and 10 µg/mL thiamine) to 9 mL of 0.5% overlay agar and vortex it before carefully pouring it on the TSA plates. A neutralization control and a negative control were also plated, and they were incubated overnight in 37 °C incubators. All bacteriophage tests were performed in triplicates.

#### 2.4.2. Human Coronavirus-229E

Huh-7.5 cells were cultured at 37 °C and 5% CO_2_ with DMEM medium supplemented with 10% (*v*/*v*) fetal bovine serum (Sigma-Aldrich, F7524-500ML, Burlington, MA, USA) and 1% (*v*/*v*) penicillin–streptomycin. Huh-7.5 cells were seeded with a density of 500,000 cells/well in 6-well plates. The suspension inactivation assay of coronavirus-229E was conducted with CNO and CNE. Freeze-thawed 100 μL of viral culture was mixed in the flipper with 100 μL of the inactivation agents for varying reaction periods (5, 15, and 30 min). In addition to the disinfection samples, a negative control (100 μL virus + 100 μL PBS) and neutralization control (100 μL disinfectant+ 1080 μL of FBS) were also analyzed. All the samples were neutralized with 10% *v/v* FBS. Measurements were conducted in triplicates. Following that, the individual controls were serially diluted up to 6 dilutions, and 300 μL of the sample was added to each well in the 6 well plates. The samples were sealed on top of the wells by adding 2 mL of 1:1 dilution of 2.4% Avicel solution and 2× DMEM and the plates were placed in a 33 °C and 5% CO_2_ incubator for 4 days to allow plaque formation. On the 4th day, 3.7% formaldehyde solution was prepared by diluting it in regular media. The plates were removed from the incubator and the Avicel solution was slowly removed and replaced by adding 2 mL of the formaldehyde solution. The formaldehyde helps to interlink the cells and makes sure it does not get affected during the following staining process. After an incubation period of 1 h, the plates were again removed from the incubator and washed with cell-grade water to remove any excess formaldehyde solution followed by staining with 200 μL of crystal violet per well. The samples were then incubated for 30–60 min with gentle shaking to stain the plaques. Water was used to remove the crystal violet. The plaques were counted, and the PFU/mL was calculated.

##### Cytotoxicity Assay

Different concentrations of CNO and CNE were added to 96-well cell culture plates containing a monolayer of HUH 7.5 cells and incubated at 33 °C and 5% CO_2_ incubator for 4 days to allow plaque formation. Cytotoxicity effects were determined by visual inspection under the optical microscope.

##### Statistical Analysis

The experimental results from triplicate measurements were expressed as mean values and standard deviation (mean ± SD). Turkey-test, one-way-ANOVA, and multiple comparison Turkey-tests were performed using GraphPad Prism version 9.3.1 (350) for Mac OS X, GraphPad Software LLC, San Diego, CA, USA, (www.graphpad.com). A *p*-value < 0.05 was considered significant.

## 3. Results

### 3.1. Characteristics and Stability of the Nanoemulsion

**Figure 3:**
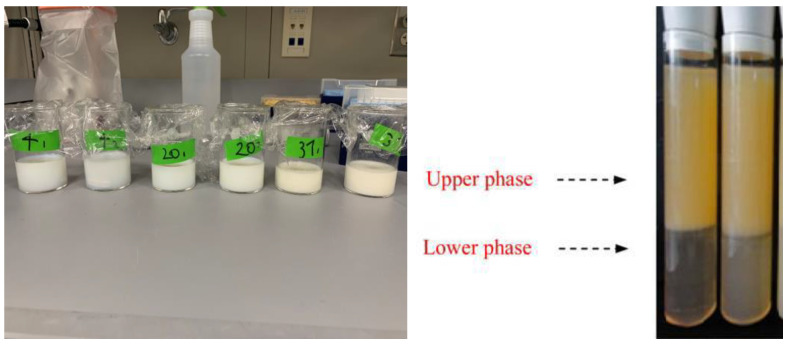
Photo images of the nanoemulsion stored at different temperatures over time. The image on the left reports the change in the color as the temperature increases from 4 °C to 37 °C; the image on the right reports a close-up sample of phase separation of the emulsion at 37 °C.

Changes in the physical characteristics of the nanoemulsions were analyzed when they were stored in incubators at three different temperatures: 4, 20, and 37 °C, which were selected to reflect refrigeration, room, and body temperatures, respectively. As the storage temperature and time increased, the color and opacity of the nanoemulsions increased (Figure 3). Additionally, visible separation of the water and oil phases was observed in the nanoemulsions after the 4th day when stored at 37 °C.

**Figure 4:**
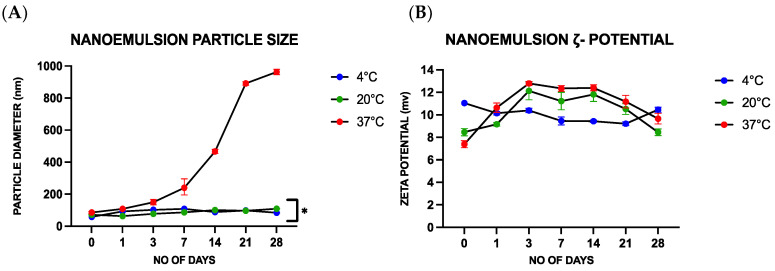
(**A**) Nanoemulsion particle size diameter (nm) recorded when stored at 4 °C, 20 °C and 37 °C for a period ranging from 0 to 28 days. (**B**) Nanoemulsion ζ-Potential recorded when stored at 4 °C, 20 °C and 37 °C for a period ranging from 0 to 28 days. *—*p*-value < 0.05.

Experiments were, therefore, carried out to optimize the composition of the nanoemulsions so as to enhance their stability. Initially, the impact of varying the cinnamaldehyde oil concentration within the nanoemulsions (0.5%, 1%, 1.5%, 2%, 2.5%, 3%, 3.5%) on their properties was investigated. The cinnamaldehyde concentration present in the nanoemulsion was 5.55 μg/mL, 11.1 μg/mL, 16.6 μg/mL, 22.2 μg/mL, 27.7 μg/mL, 33.3 μg/mL and 38.8 μg/mL, respectively. The mean particle diameter increased with increasing oil concentration, particularly when the cinnamaldehyde concentration exceeded 27.7 μg/mL. Notably, at a cinnamaldehyde concentration of 38.8 μg/mL, the measured particle size ranged from 569.8 to 729.6 nm. Hence, it was imperative to maintain the concentration of CNO at or below 27.7 μg/mL. The upper limit of this concentration was chosen to conduct a shelf-life study by storing the nanoemulsion at a range of temperatures (4, 20, and 37 °C) for periods ranging from 0 to 28 days. There was also a steep increase in the mean particle diameter from 150.16 ± 16.5 nm on the 3rd day to around 962.88 ± 16.3 nm on the 28th day when the samples were stored at 37 °C (Figure 4A). Notably, this steep increase in particle size was only observed in the samples stored at 37 °C, and no significant changes were noticed when the nanoemulsions were stored at 4 or 20 °C. However, a slight increase in droplet size was observed when the nanoemulsions were stored at 20 °C, but this was not significant. These results suggest that the storage temperature is important for maintaining the stability of the nanoemulsions prepared in this study. The increase in droplet size observed at higher temperatures may have been due to coalescence or Ostwald ripening. The polydispersity index (PDI = 0.2–0.3) remained relatively low during storage for the nanoemulsions kept at lower temperatures (4 and 20 °C), which indicates that the droplets remained relatively uniform in dimensions [24]. In drug delivery applications, using lipid-based carriers, such as nanoliposome formulations, a PDI of 0.3 and below is considered acceptable and a homogenous population [25]

The antimicrobial activity of ionic surfactants such as CTAB has been attributed to their ability to be incorporated into the lipid membrane of microbial cells, thereby disrupting normal cellular function [26]. The surfaces of microorganisms are typically negatively charged, and therefore positively charged oil droplets would be expected to be electrostatically attracted to their surfaces [20]. The electrical charge (ζ-potential) characteristics of the oil droplets in nanoemulsions containing different amounts of CTAB were therefore measured. The lowest concentration of CTAB used (0.02%) was enough to result in a charge ranging between +10–12 mV. However, an increase in CTAB concentration resulted in an undesirable rise in the droplet size; e.g., the mean particle diameter increased to 520 ± 13 nm at 0.2% CTAB. Thus, a lower concentration of CTAB was used to formulate the nanoemulsions. The ζ-potential values of the nanoemulsions were also measured during storage at different temperatures, and no drastic changes were observed (Figure 4B), being 11.11 ± 0.09, 11.34 ± 0.24, and 12.15 ± 0.13 mV when stored at 4, 20, and 37 °C, respectively.

### 3.2. Antiviral Activity Against MS2 Bacteriophage

The antiviral activity of the CNO and CNE samples was measured against MS2 Bacteriophage, a human norovirus surrogate. The inactivation studies were conducted for a range of concentrations (0.5%, 1%, 1.5%, 2%, 2.5%, 3%, 3.5%) of cinnamaldehyde after 60 min treatment, as well as for a range of treatment times of 5, 15, 30, and 60 min for 2.5% cinnamaldehyde (27.7 ug/mL).

#### 3.2.1. CNO Treatment

**Figure 5:**
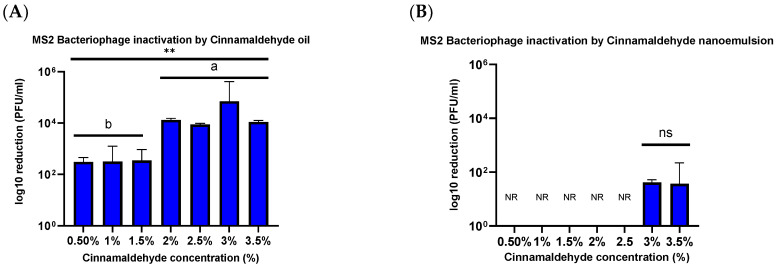
(**A**) MS2 bacteriophage microbial reduction post-treatment with a range of cinnamaldehyde oil (0.5–3.5%). (**B**) MS2 Bacteriophage microbial reduction post-treatment with a range of cinnamaldehyde nanoemulsion (0.5–3.5%) treated for 60 min. NR—no reduction, NS—no significant difference, **—determines statistical significance (*p* < 0.05). Different letters indicate statistical differences.

When treated with bulk essential oil for 60 min, viral inactivation depended on oil concentration, being 2.78 ± 0.34, 4.12 ± 0.10, 3.94 ± 0.04, 4.84 ± 0.70, and 4.04 ± 0.05 PFU/mL log10 reduction at 0.5, 2, 2.5, 3, and 3.5% oil, respectively (Figure 5A). At lower reaction time periods, no significant reduction was observed when MS2 was treated for 5, 15, and 30 min.

#### 3.2.2. CNE Treatment

When the CNE formulations with cinnamaldehyde at equal concentrations to that of the CNO were exposed to MS2, significantly lower viral reductions were observed for the emulsified oil than for the bulk oil, ranging from no reduction observed with the lowest cinnamaldehyde concentration (0.5%) to a maximum of 1.54 ± 0.08 PFU/mL log reduction for the highest concentration (3.5%) (Figure 5B). However, it should be noted that CNE formulations with 3% and 3.5% cinnamaldehyde exhibited quite large particle sizes, 200 nm, and 600 nm, respectively, that could comprise emulsion stability.

### 3.3. Antiviral Activity of CNO and CNE Against Human Coronavirus-229E

**Figure 6:**
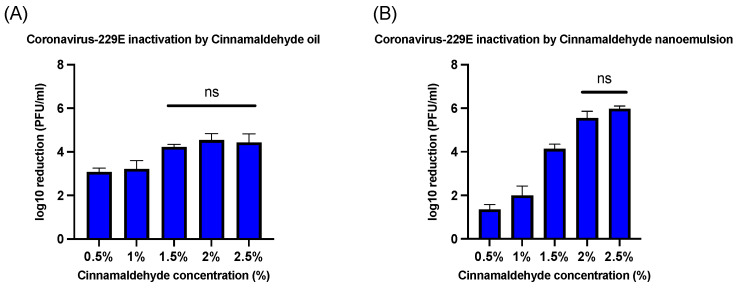
(**A**) Human coronavirus-229E microbial reduction post-treatment with a range of CNO (0.5–2.5%). (**B**) Human coronavirus-229E microbial reduction post-treatment with a range of CNE (0.5–3.5%). NS—no significant difference.

**Figure 7:**
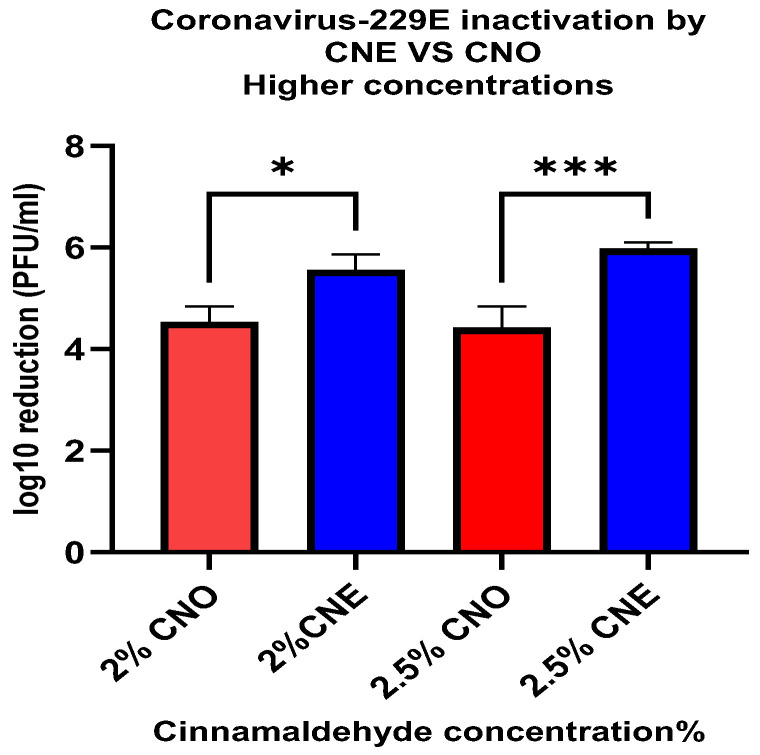
Human coronavirus-229E microbial reduction post-treatment with 2% CNO, 2.5% CNE, 2.5% CNO, and 2.5% CNE. *—*p*-value < 0.05, ***—*p*-value < 0.001.

Both CNO and CNE exhibited a higher degree of antiviral activity against coronavirus-229E than against bacteriophage MS2 (Figure 6). When CNO was exposed to coronavirus-229E for 1 min, there was at least a 3-log reduction at all concentrations; however, there was no significant difference above concentration 1.5% (Figure 6A). In addition, when CNE was exposed to 229E for 1 min, reductions of 1.35 ± 0.23, 2 ± 0.43, 4.13 ± 0.21, 5.56 ± 0.3, and 5.98 ± 0.12 PFU/mL log10 reductions were observed for cinnamaldehyde concentrations of 0.5, 1, 1.5, 2, and 2.5%, respectively (Figure 6B). Interestingly, there was an increase in the efficiency of cinnamaldehyde against coronavirus-229E when incorporated into the nanoemulsion at higher concentrations such as 2% and 2.5%. The increase in efficiency was significantly higher at a 2.5% concentration of CNE when compared to 2.5% CNO as seen in Figure 7. This suggests that CNE may enhance the antiviral efficacy of cinnamaldehyde at higher concentrations (2–2.5%).

## 4. Discussion

Essential oils (EOs) are aromatic, oil-like volatile substances present in plant materials such as fruits, bark, seeds, pulp, peel, root, and whole plant. The use of synthetic antimicrobial compounds as food preservatives has raised consumers’ concerns, since they present numerous toxicological difficulties and may not be safe for human consumption. Hence, over the last two decades, natural antimicrobial agents such as EOs have received strong interest from the scientific community, owing to their unique physicochemical properties and diverse biological activities [27].

Researchers have demonstrated that cinnamaldehyde has a strong antibacterial effect against *Staphylococcus epidermidis*, *Staphylococcus aureus*, and *Aeromonas hydrophila* by causing cell membrane distortion, leakage, and polarization of the cytoplasmic content [11,12]. Several reports have documented that cinnamon oil and its major constituent, cinnamaldehyde, exhibit various biological activities in vitro, such as antibacterial activity [28], induction of apoptosis via reactive oxygen species [29], and inhibition of nitric oxide synthesis [30]. However, less is known about its antiviral activity. Many antimicrobials are less effective at inactivating non-enveloped viruses (such as the human norovirus) than bacteria or fungi because they lack a lipid envelope [15,31] Instead, non-enveloped viruses have a protein capsid that encloses their genetic material and protects it from environmental stresses. In contrast, enveloped viruses have an outer lipid envelope that is typically more fragile and susceptible to environmental stresses [32,33]. The results of the current study are consistent with this difference in the structure of different viruses. There was a relatively small decrease (<1.54 ± 0.08 PFU/mL log reduction) in the viability of the MS2 bacteriophage when treated with 2.5% and 3% CNE for 1 h. However, there was a relatively high decrease (5.98 ± 0.12 PFU/mL log reduction) in viability when HuCoV-229E was treated with 2.5% CNE for 1 min.

Antimicrobial nanoemulsions contain small oil droplets that are coated by a layer of surfactant. These surfactant-coated oil droplets can rapidly move through aqueous solutions and fuse with bacterial cell walls or viral envelopes, thereby destabilizing them, which leads to the disruption and deactivation of the microorganisms. Norovirus has a protein capsid that is generally resistant to lipophilic inactivation agents (e.g., quaternary ammonium compounds) and solvents (e.g., alcohols) [17,34]. While noroviruses are non-enveloped, they do have patches of hydrophobic amino acid residues in their major capsid protein that are critical to folding and maintaining the icosahedral contacts needed to form the norovirus capsid. In addition, the cationic oil droplets in the nanoemulsions will be electrostatically attracted to the anionic capsid of the virus, which may enhance their ability to deliver the antiviral agents to the site of action. Hence, the main goal of this research was to evaluate the efficiency of CNE against negatively charged protein capsids present in non-enveloped virus human norovirus surrogate bacteriophage MS2.

Contrary to this hypothesis, lower viral inactivation was observed when the essential oil was incorporated into the nanoemulsion droplets than for the pure essential oil. For instance, after treatment for 1 h, there was only a 1.54 ± 0.08 PFU/mL log reduction for the CNE containing the highest oil concentration but a 4.02 ± 0.102 PFU/mL log reduction for the CNO containing the same oil concentration. One explanation for the reduced efficacy of the essential oil after it was emulsified may be due to differences in the partitioning of the hydrophobic cinnamaldehyde molecules within the system. These hydrophobic antiviral molecules tend to remain inside the hydrophobic interior of the nanoemulsion droplets, rather than interact with the capsid proteins of the viruses. Thus, they only offer benefits when used with enveloped viruses. The results reported here also suggest that storing the CNEs at lower temperatures (such as 4 and 20 °C) results in an improvement in their physical stability, with less droplet growth and creaming occurring. In contrast, the nanoemulsions were unstable when stored at 37 °C, as seen by an increase in oil droplet size and visible phase separation. This instability was probably caused by droplet coalescence and Ostwald ripening. Coalescence occurs when two or more oil droplets encounter each other and fuse together, thereby leading to an increase in oil droplet size. Eventually, extensive coalescence leads to phase separation and oiling off, where a layer of bulk oil is formed on top of the system. Ostwald ripening is a process whereby the larger droplets grow at the expense of the smaller droplets due to the molecular diffusion of oil molecules through the continuous phase. Delmas et al. reported that Ostwald ripening tends to increase with increasing storage temperature, which is consistent with the reduced stability of the nanoemulsions observed at 37 °C [35]. Other researchers have also reported that nanoemulsions tend to be less stable when stored at higher temperatures. The physical instability of the nanoemulsions observed at higher temperatures led to an increase in their particle size, which may reduce their ability to interact with viruses [36]. For instance, a smaller particle size may facilitate the ability of the antiviral oil droplets to interact with and penetrate into microorganisms [37].

Alternative nanoemulsion preparation methods may be required to create nanoemulsions that are stable at higher storage temperatures [38]. The low-energy homogenization method used in the current study (spontaneous emulsification) requires the use of synthetic surfactants, which can lead to the formation of surfactant-coated oil droplets that are unstable at higher temperatures. Nanoemulsions can also be prepared using high-energy methods (such as microfluidization), which provides much greater flexibility in the choice of emulsifier used to coat the oil droplets. Consequently, it may be possible to use an emulsifier that provides better thermal stability to the oil droplets in nanoemulsions using this approach. Hence, there is still a need for further research to identify nanoemulsion formulations that are more physically stable, but that still exhibit strong antiviral efficiency.

In addition to deactivating non-enveloped viruses (such as human norovirus), it is also important to investigate the deactivation of enveloped viruses (such as SARS-CoV-2). For this reason, we examined the ability of the nanoemulsions to deactivate surrogate human coronavirus 229E. Our results showed that the emulsified essential oils exhibited much stronger antiviral activity against the enveloped virus. For instance, after just 1 min treatment using the lowest oil concentration (0.5%), there was a 1.35 ± 0.23 and 3.08 ± 0.17 PFU/mL log10 reduction for CNE and CNO, respectively. However, when the oil concentration was increased to 2.5%, a higher level of microbial reduction was observed for the CNE (5.98 ± 0.12 PFU/mL) than for the CNO (4.43 ± 0.38 PFU/mL). This result suggests that the efficiency of the essential oil as an inactivation agent against coronavirus 229E can be increased when it is incorporated in a nanoemulsion at an appropriate concentration. However, further studies should be conducted to analyze the efficacy of these essential nanoemulsions as food contact surface sanitizers under more realistic conditions.

## 5. Conclusions

Viruses continue to pose a serious health and economic threat globally and it has been estimated that they are responsible for around 2 million deaths per year [39]. Recently, there has been an emphasis on the use of natural antiviral agents because of their health and environmental benefits. However, despite their antiviral potential, there are numerous challenges limiting the efficacy of many existing antivirals such as low solubility, low bioavailability, and stability [40]. Considering these concerns, this study investigated the efficacy of a novel plant-based antiviral inactivation agent delivery system in cationic cinnamaldehyde nanoemulsions. Results demonstrated significant antiviral activity, particularly against enveloped viruses such as human coronavirus 229E (HuCoV-229E), with a high reduction in viral infectivity observed at optimal nanoemulsion concentrations. However, the effectiveness varied amongst the two different viruses tested, with non-enveloped viruses like norovirus surrogate phage MS2 showing less susceptibility than suspended oil alone. This suggests that such a delivery system is likely only efficacious against enveloped viruses, like coronaviruses. It also suggests that nanoemulsion composition and formulation play a critical role in antiviral efficacy, potentially influencing interactions with viral envelope structure or capsid proteins. Further studies should be conducted to evaluate the efficacy of different nanoemulsions against a wider range of enveloped viruses. This study focused on conducting antiviral studies in suspension. Further studies can be conducted to evaluate the inactivation efficacy by conducting surface studies. Additionally, further studies can be conducted to investigate the efficiency of the inactivation agent on surfaces by varying the surface reaction time. From the results, it was seen that after treatment of MS2 bacteriophage for 1 h, the reduction in numbers was significantly less for the CNE than for the CNO. For instance, the log reductions in MS2 numbers were 4.02 ± 0.10 and 2.78 ± 0.34 PFU/mL after treatment with 3.5% and 0.5% of CNO, whereas they were only 1.54 ± 0.08 and 0 PFU/mL after treatment with the equivalent CNE, respectively. Hence, a range of time and concentration can be evaluated to further understand the efficiency of CNE on surfaces, specifically against MS2 Bacteriophage. Airborne microorganisms are one of the major concerns, hence further studies can also be conducted to evaluate the efficiency of CNE in aerosolized environments. This work informs the development of the next generation of inactivation agents with enhanced efficacy against viruses with reduced chemical residues and harmful environmental effects.

## Data Availability

The data presented in this study are available on request from the corresponding author. The data are not publicly available due to privacy restrictions.

## Abbreviations

The following abbreviations are used in this manuscript:

CNO	Cinnamaldehyde oil
CNE	Cinnamaldehyde nanoemulsion
HuCoV-229E	Human coronavirus-229E
CTAB	Hexadecyltrimethylammonium bromide
FBS	Fetal bovine serum
DMEM	Dulbecco’s modified eagle medium
